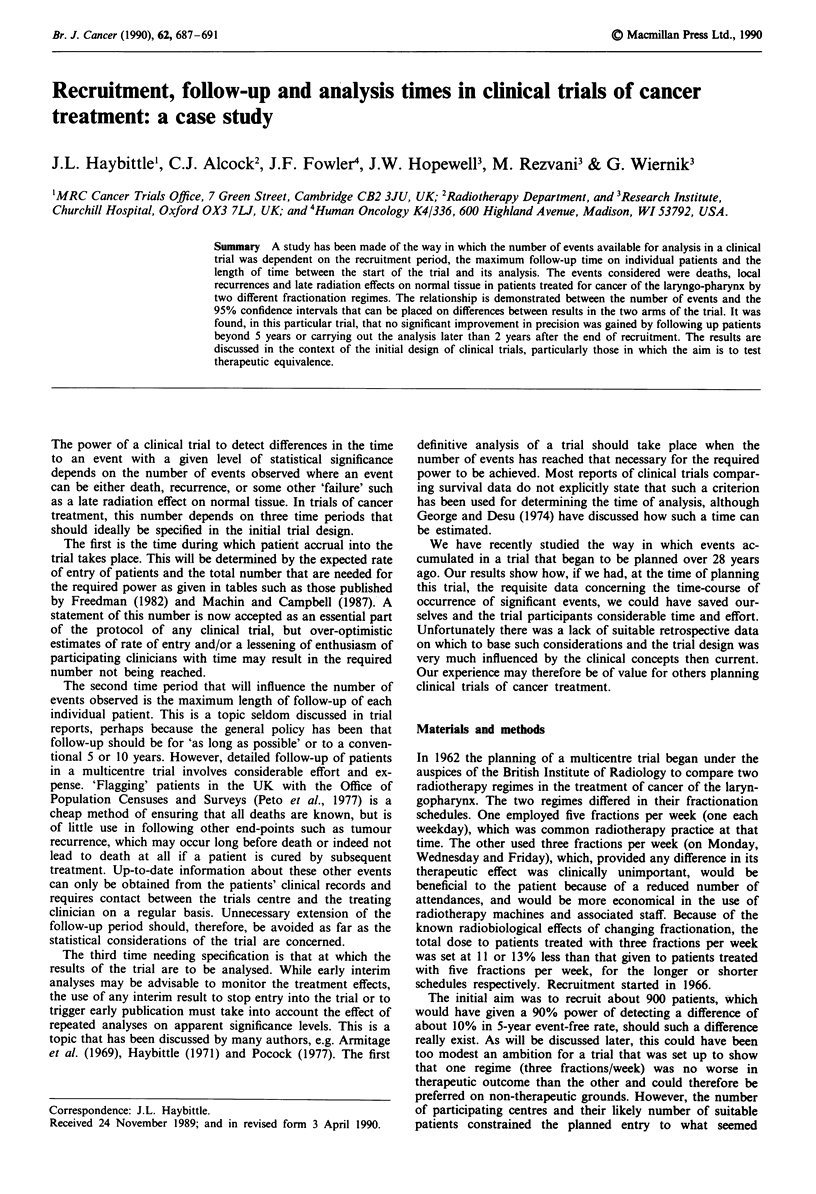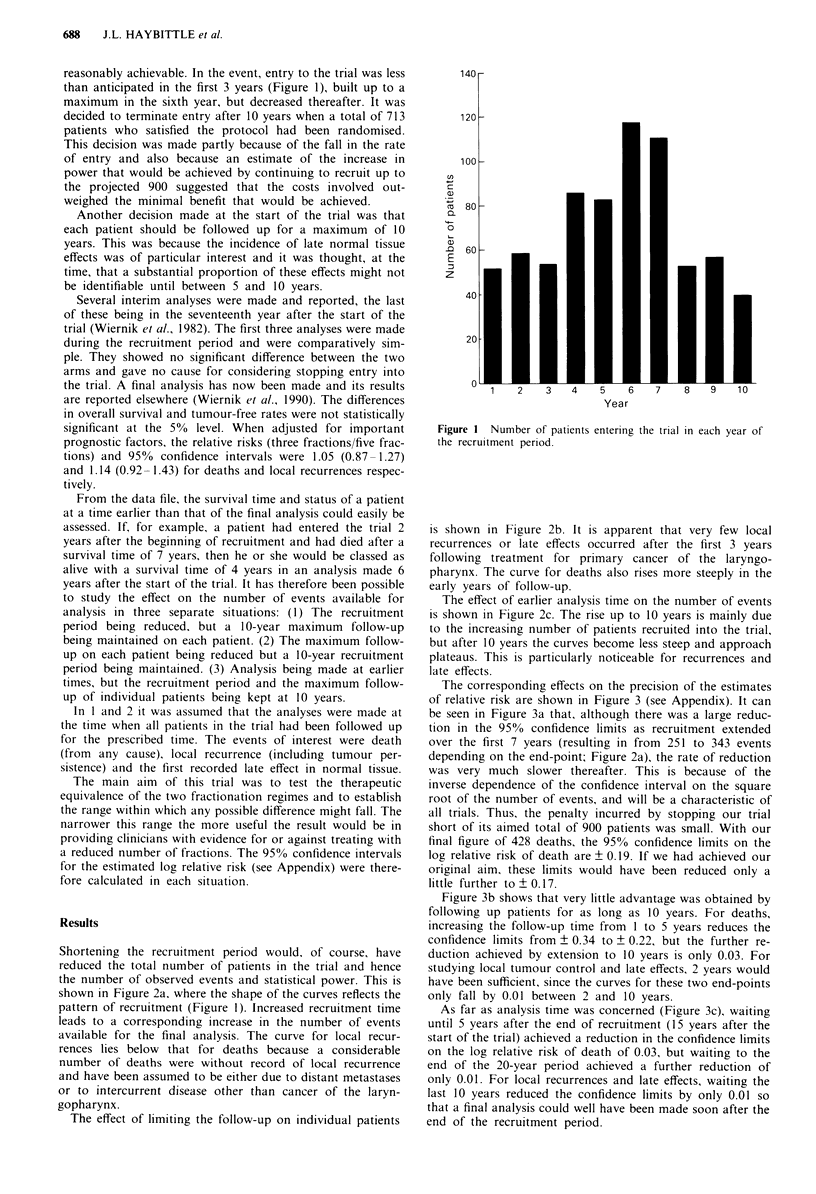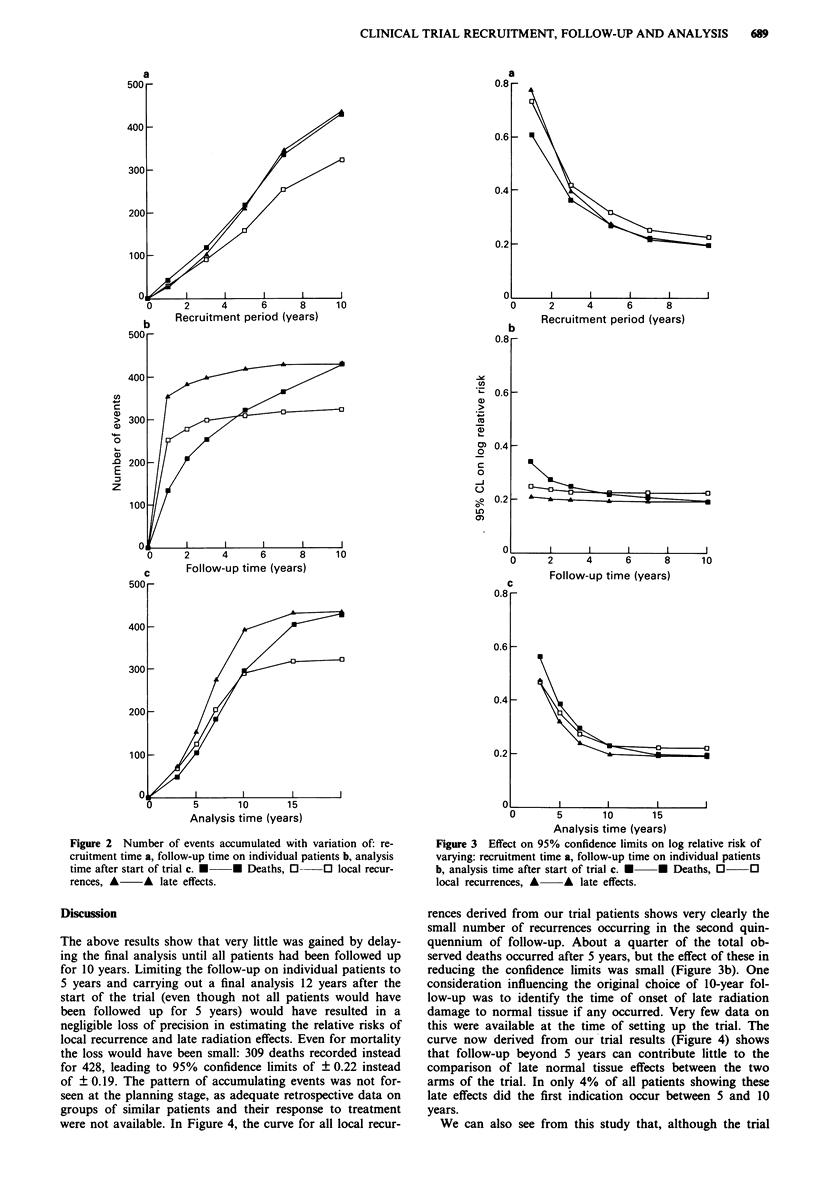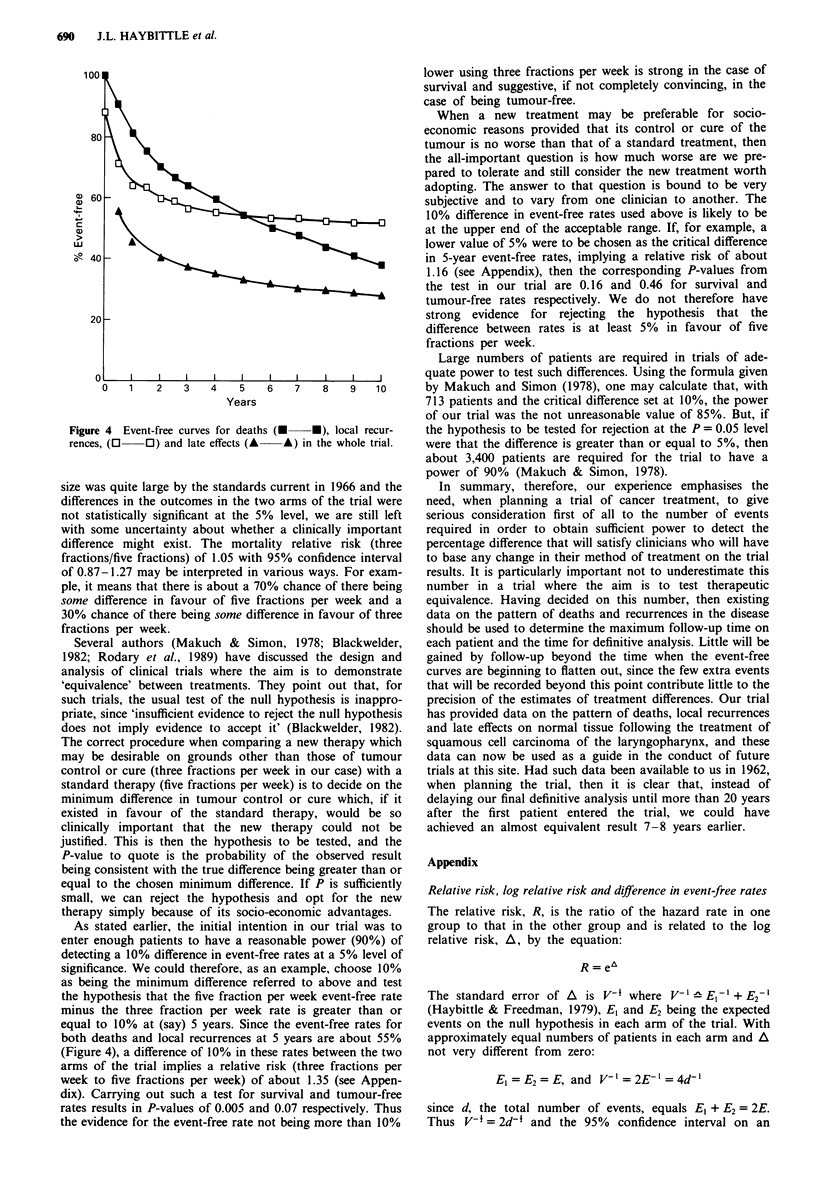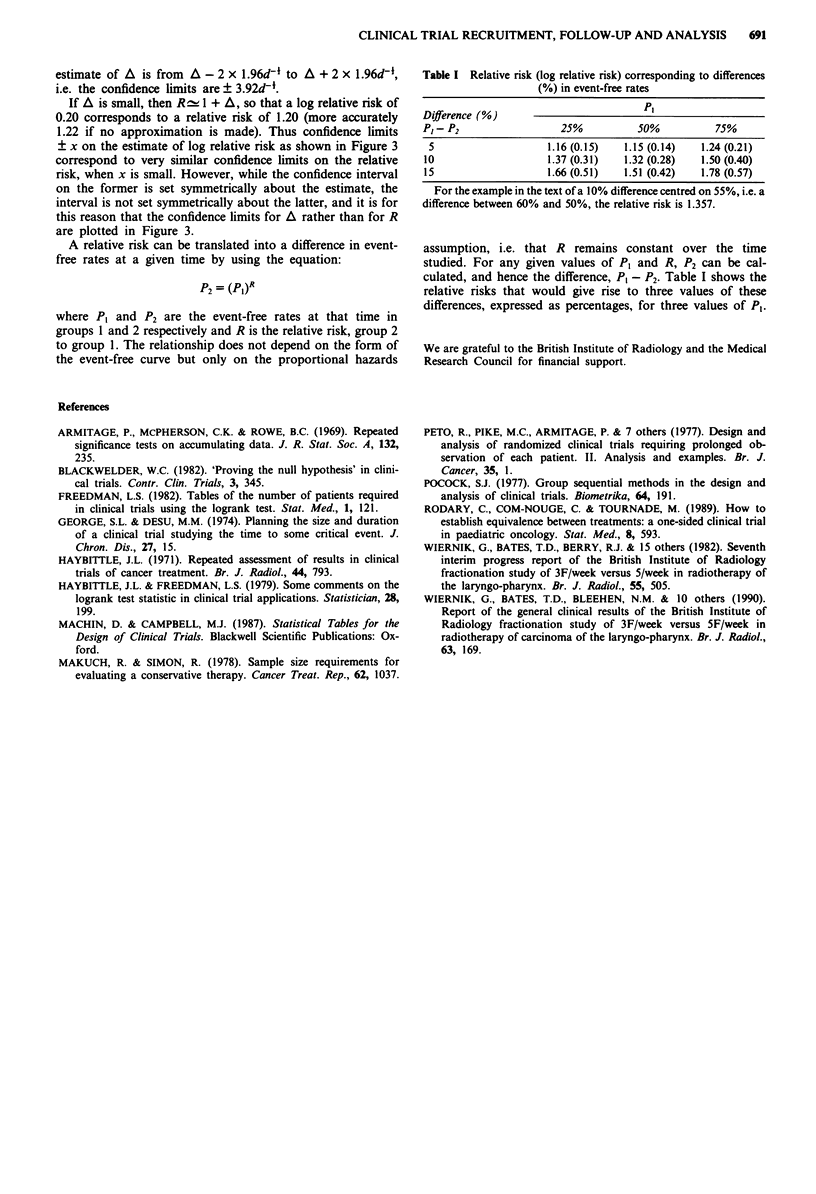# Recruitment, follow-up and analysis times in clinical trials of cancer treatment: a case study.

**DOI:** 10.1038/bjc.1990.358

**Published:** 1990-10

**Authors:** J. L. Haybittle, C. J. Alcock, J. F. Fowler, J. W. Hopewell, M. Rezvani, G. Wiernik

**Affiliations:** MRC Cancer Trials Office, Cambridge, UK.

## Abstract

A study has been made of the way in which the number of events available for analysis in a clinical trial was dependent on the recruitment period, the maximum follow-up time on individual patients and the length of time between the start of the trial and its analysis. The events considered were deaths, local recurrences and late radiation effects on normal tissue in patients treated for cancer of the laryngo-pharynx by two different fractionation regimes. The relationship is demonstrated between the number of events and the 95% confidence intervals that can be placed on differences between results in the two arms of the trial. It was found, in this particular trial, that no significant improvement in precision was gained by following up patients beyond 5 years or carrying out the analysis later than 2 years after the end of recruitment. The results are discussed in the context of the initial design of clinical trials, particularly those in which the aim is to test therapeutic equivalence.


Br. J. Cancer (1990), 62, 687-691

© Macmillan Press Ltd., 1990

### Recruitment, follow-up and analysis times in clinical trials of cancer treatment: a case study

J.L. Haybittle[1], C.J. Alcock[2], J.F. Fowler[4], J.W. Hopewell[3], M. Rezvani[3] & G. Wiernik[3]

[1]MRC Cancer Trials Office, 7 Green Street, Cambridge CB2 3JU, UK; [2]Radiotherapy Department, and [3]Research Institute, Churchill Hospital, Oxford OX3 7LJ, UK; and [4]Human Oncology K4/336, 600 Highland Avenue, Madison, WI 53792, USA.

**Summary** A study has been made of the way in which the number of events available for analysis in a clinical trial was dependent on the recruitment period, the maximum follow-up time on individual patients and the length of time between the start of the trial and its analysis. The events considered were deaths, local recurrences and late radiation effects on normal tissue in patients treated for cancer of the laryngo-pharynx by two different fractionation regimes. The relationship is demonstrated between the number of events and the 95% confidence intervals that can be placed on differences between results in the two arms of the trial. It was found, in this particular trial, that no significant improvement in precision was gained by following up patients beyond 5 years or carrying out the analysis later than 2 years after the end of recruitment. The results are discussed in the context of the initial design of clinical trials, particularly those in which the aim is to test therapeutic equivalence.

The power of a clinical trial to detect differences in the time to an event with a given level of statistical significance depends on the number of events observed where an event can be either death, recurrence, or some other 'failure' such as a late radiation effect on normal tissue. In trials of cancer treatment, this number depends on three time periods that should ideally be specified in the initial trial design.

The first is the time during which patient accrual into the trial takes place. This will be determined by the expected rate of entry of patients and the total number that are needed for the required power as given in tables such as those published by Freedman (1982) and Machin and Campbell (1987). A statement of this number is now accepted as an essential part of the protocol of any clinical trial, but over-optimistic estimates of rate of entry and/or a lessening of enthusiasm of participating clinicians with time may result in the required number not being reached.

The second time period that will influence the number of events observed is the maximum length of follow-up of each individual patient. This is a topic seldom discussed in trial reports, perhaps because the general policy has been that follow-up should be for 'as long as possible' or to a conventional 5 or 10 years. However, detailed follow-up of patients in a multicentre trial involves considerable effort and expense. 'Flagging' patients in the UK with the Office of Population Censuses and Surveys (Peto *et al.*, 1977) is a cheap method of ensuring that all deaths are known, but is of little use in following other end-points such as tumour recurrence, which may occur long before death or indeed not lead to death at all if a patient is cured by subsequent treatment. Up-to-date information about these other events can only be obtained from the patients' clinical records and requires contact between the trials centre and the treating clinician on a regular basis. Unnecessary extension of the follow-up period should, therefore, be avoided as far as the statistical considerations of the trial are concerned.

The third time needing specification is that at which the results of the trial are to be analysed. While early interim analyses may be advisable to monitor the treatment effects, the use of any interim result to stop entry into the trial or to trigger early publication must take into account the effect of repeated analyses on apparent significance levels. This is a topic that has been discussed by many authors, e.g. Armitage *et al.* (1969), Haybittle (1971) and Pocock (1977). The first definitive analysis of a trial should take place when the number of events has reached that necessary for the required power to be achieved. Most reports of clinical trials comparing survival data do not explicitly state that such a criterion has been used for determining the time of analysis, although George and Desu (1974) have discussed how such a time can be estimated.

We have recently studied the way in which events accumulated in a trial that began to be planned over 28 years ago. Our results show how, if we had, at the time of planning this trial, the requisite data concerning the time-course of occurrence of significant events, we could have saved ourselves and the trial participants considerable time and effort. Unfortunately there was a lack of suitable retrospective data on which to base such considerations and the trial design was very much influenced by the clinical concepts then current. Our experience may therefore be of value for others planning clinical trials of cancer treatment.

### Materials and methods

In 1962 the planning of a multicentre trial began under the auspices of the British Institute of Radiology to compare two radiotherapy regimes in the treatment of cancer of the laryngopharynx. The two regimes differed in their fractionation schedules. One employed five fractions per week (one each weekday), which was common radiotherapy practice at that time. The other used three fractions per week (on Monday, Wednesday and Friday), which, provided any difference in its therapeutic effect was clinically unimportant, would be beneficial to the patient because of a reduced number of attendances, and would be more economical in the use of radiotherapy machines and associated staff. Because of the known radiobiological effects of changing fractionation, the total dose to patients treated with three fractions per week was set at 11 or 13% less than that given to patients treated with five fractions per week, for the longer or shorter schedules respectively. Recruitment started in 1966.

The initial aim was to recruit about 900 patients, which would have given a 90% power of detecting a difference of about 10% in 5-year event-free rate, should such a difference really exist. As will be discussed later, this could have been too modest an ambition for a trial that was set up to show that one regime (three fractions/week) was no worse in therapeutic outcome than the other and could therefore be preferred on non-therapeutic grounds. However, the number of participating centres and their likely number of suitable patients constrained the planned entry to what seemed

Correspondence: J.L. Haybittle.
Received 24 November 1989; and in revised form 3 April 1990.

reasonably achievable. In the event, entry to the trial was less than anticipated in the first 3 years (Figure 1), built up to a maximum in the sixth year, but decreased thereafter. It was decided to terminate entry after 10 years when a total of 713 patients who satisfied the protocol had been randomised. This decision was made partly because of the fall in the rate of entry and also because an estimate of the increase in power that would be achieved by continuing to recruit up to the projected 900 suggested that the costs involved outweighed the minimal benefit that would be achieved.

Another decision made at the start of the trial was that each patient should be followed up for a maximum of 10 years. This was because the incidence of late normal tissue effects was of particular interest and it was thought, at the time, that a substantial proportion of these effects might not be identifiable until between 5 and 10 years.

Several interim analyses were made and reported, the last of these being in the seventeenth year after the start of the trial (Wiernik et al., 1982). The first three analyses were made during the recruitment period and were comparatively simple. They showed no significant difference between the two arms and gave no cause for considering stopping entry into the trial. A final analysis has now been made and its results are reported elsewhere (Wiernik et al., 1990). The differences in overall survival and tumour-free rates were not statistically significant at the 5% level. When adjusted for important prognostic factors, the relative risks (three fractions/five fractions) and 95% confidence intervals were 1.05 (0.87–1.27) and 1.14 (0.92–1.43) for deaths and local recurrences respectively.

From the data file, the survival time and status of a patient at a time earlier than that of the final analysis could easily be assessed. If, for example, a patient had entered the trial 2 years after the beginning of recruitment and had died after a survival time of 7 years, then he or she would be classed as alive with a survival time of 4 years in an analysis made 6 years after the start of the trial. It has therefore been possible to study the effect on the number of events available for analysis in three separate situations: (1) The recruitment period being reduced, but a 10-year maximum follow-up being maintained on each patient. (2) The maximum follow-up on each patient being reduced but a 10-year recruitment period being maintained. (3) Analysis being made at earlier times, but the recruitment period and the maximum follow-up of individual patients being kept at 10 years.

In 1 and 2 it was assumed that the analyses were made at the time when all patients in the trial had been followed up for the prescribed time. The events of interest were death (from any cause), local recurrence (including tumour persistence) and the first recorded late effect in normal tissue.

The main aim of this trial was to test the therapeutic equivalence of the two fractionation regimes and to establish the range within which any possible difference might fall. The narrower this range the more useful the result would be in providing clinicians with evidence for or against treating with a reduced number of fractions. The 95% confidence intervals for the estimated log relative risk (see Appendix) were therefore calculated in each situation.

### Results

Shortening the recruitment period would, of course, have reduced the total number of patients in the trial and hence the number of observed events and statistical power. This is shown in Figure 2a, where the shape of the curves reflects the pattern of recruitment (Figure 1). Increased recruitment time leads to a corresponding increase in the number of events available for the final analysis. The curve for local recurrences lies below that for deaths because a considerable number of deaths were without record of local recurrence and have been assumed to be either due to distant metastases or to intercurrent disease other than cancer of the laryngopharynx.

The effect of limiting the follow-up on individual patients is shown in Figure 2b. It is apparent that very few local recurrences or late effects occurred after the first 3 years following treatment for primary cancer of the laryngopharynx. The curve for deaths also rises more steeply in the early years of follow-up.

The effect of earlier analysis time on the number of events is shown in Figure 2c. The rise up to 10 years is mainly due to the increasing number of patients recruited into the trial, but after 10 years the curves become less steep and approach plateaus. This is particularly noticeable for recurrences and late effects.

The corresponding effects on the precision of the estimates of relative risk are shown in Figure 3 (see Appendix). It can be seen in Figure 3a that, although there was a large reduction in the 95% confidence limits as recruitment extended over the first 7 years (resulting in from 251 to 343 events depending on the end-point; Figure 2a), the rate of reduction was very much slower thereafter. This is because of the inverse dependence of the confidence interval on the square root of the number of events, and will be a characteristic of all trials. Thus, the penalty incurred by stopping our trial short of its aimed total of 900 patients was small. With our final figure of 428 deaths, the 95% confidence limits on the log relative risk of death are ± 0.19. If we had achieved our original aim, these limits would have been reduced only a little further to ± 0.17.

Figure 3b shows that very little advantage was obtained by following up patients for as long as 10 years. For deaths, increasing the follow-up time from 1 to 5 years reduces the confidence limits from ± 0.34 to ± 0.22, but the further reduction achieved by extension to 10 years is only 0.03. For studying local tumour control and late effects, 2 years would have been sufficient, since the curves for these two end-points only fall by 0.01 between 2 and 10 years.

As far as analysis time was concerned (Figure 3c), waiting until 5 years after the end of recruitment (15 years after the start of the trial) achieved a reduction in the confidence limits on the log relative risk of death of 0.03, but waiting to the end of the 20-year period achieved a further reduction of only 0.01. For local recurrences and late effects, waiting the last 10 years reduced the confidence limits by only 0.01 so that a final analysis could well have been made soon after the end of the recruitment period.

**Figure 1** Number of patients entering the trial in each year of the recruitment period.

**Figure 2** Number of events accumulated with variation of: recruitment time **a**, follow-up time on individual patients **b**, analysis time after start of trial **c**. ■——■ Deaths, □——□ local recurrences, ▲——▲ late effects.

**Figure 3** Effect on 95% confidence limits on log relative risk of varying: recruitment time **a**, follow-up time on individual patients **b**, analysis time after start of trial **c**. ■——■ Deaths, □——□ local recurrences, ▲——▲ late effects.

### Discussion

The above results show that very little was gained by delaying the final analysis until all patients had been followed up for 10 years. Limiting the follow-up on individual patients to 5 years and carrying out a final analysis 12 years after the start of the trial (even though not all patients would have been followed up for 5 years) would have resulted in a negligible loss of precision in estimating the relative risks of local recurrence and late radiation effects. Even for mortality the loss would have been small: 309 deaths recorded instead for 428, leading to 95% confidence limits of $\pm 0.22$ instead of $\pm 0.19$. The pattern of accumulating events was not forseen at the planning stage, as adequate retrospective data on groups of similar patients and their response to treatment were not available. In Figure 4, the curve for all local recurrences derived from our trial patients shows very clearly the small number of recurrences occurring in the second quinquennium of follow-up. About a quarter of the total observed deaths occurred after 5 years, but the effect of these in reducing the confidence limits was small (Figure 3b). One consideration influencing the original choice of 10-year follow-up was to identify the time of onset of late radiation damage to normal tissue if any occurred. Very few data on this were available at the time of setting up the trial. The curve now derived from our trial results (Figure 4) shows that follow-up beyond 5 years can contribute little to the comparison of late normal tissue effects between the two arms of the trial. In only 4% of all patients showing these late effects did the first indication occur between 5 and 10 years.

We can also see from this study that, although the trial

**Figure 4** Event-free curves for deaths (■——■), local recurrences, (□——□) and late effects (▲——▲) in the whole trial.

size was quite large by the standards current in 1966 and the differences in the outcomes in the two arms of the trial were not statistically significant at the 5% level, we are still left with some uncertainty about whether a clinically important difference might exist. The mortality relative risk (three fractions/five fractions) of 1.05 with 95% confidence interval of 0.87–1.27 may be interpreted in various ways. For example, it means that there is about a 70% chance of there being *some* difference in favour of five fractions per week and a 30% chance of there being *some* difference in favour of three fractions per week.

Several authors (Makuch & Simon, 1978; Blackwelder, 1982; Rodary *et al.*, 1989) have discussed the design and analysis of clinical trials where the aim is to demonstrate 'equivalence' between treatments. They point out that, for such trials, the usual test of the null hypothesis is inappropriate, since 'insufficient evidence to reject the null hypothesis does not imply evidence to accept it' (Blackwelder, 1982). The correct procedure when comparing a new therapy which may be desirable on grounds other than those of tumour control or cure (three fractions per week in our case) with a standard therapy (five fractions per week) is to decide on the minimum difference in tumour control or cure which, if it existed in favour of the standard therapy, would be so clinically important that the new therapy could not be justified. This is then the hypothesis to be tested, and the $P$-value to quote is the probability of the observed result being consistent with the true difference being greater than or equal to the chosen minimum difference. If $P$ is sufficiently small, we can reject the hypothesis and opt for the new therapy simply because of its socio-economic advantages.

As stated earlier, the initial intention in our trial was to enter enough patients to have a reasonable power (90%) of detecting a 10% difference in event-free rates at a 5% level of significance. We could therefore, as an example, choose 10% as being the minimum difference referred to above and test the hypothesis that the five fraction per week event-free rate minus the three fraction per week rate is greater than or equal to 10% at (say) 5 years. Since the event-free rates for both deaths and local recurrences at 5 years are about 55% (Figure 4), a difference of 10% in these rates between the two arms of the trial implies a relative risk (three fractions per week to five fractions per week) of about 1.35 (see Appendix). Carrying out such a test for survival and tumour-free rates results in $P$-values of 0.005 and 0.07 respectively. Thus the evidence for the event-free rate not being more than 10% lower using three fractions per week is strong in the case of survival and suggestive, if not completely convincing, in the case of being tumour-free.

When a new treatment may be preferable for socio-economic reasons provided that its control or cure of the tumour is no worse than that of a standard treatment, then the all-important question is how much worse are we prepared to tolerate and still consider the new treatment worth adopting. The answer to that question is bound to be very subjective and to vary from one clinician to another. The 10% difference in event-free rates used above is likely to be at the upper end of the acceptable range. If, for example, a lower value of 5% were to be chosen as the critical difference in 5-year event-free rates, implying a relative risk of about 1.16 (see Appendix), then the corresponding $P$-values from the test in our trial are 0.16 and 0.46 for survival and tumour-free rates respectively. We do not therefore have strong evidence for rejecting the hypothesis that the difference between rates is at least 5% in favour of five fractions per week.

Large numbers of patients are required in trials of adequate power to test such differences. Using the formula given by Makuch and Simon (1978), one may calculate that, with 713 patients and the critical difference set at 10%, the power of our trial was the not unreasonable value of 85%. But, if the hypothesis to be tested for rejection at the $P = 0.05$ level were that the difference is greater than or equal to 5%, then about 3,400 patients are required for the trial to have a power of 90% (Makuch & Simon, 1978).

In summary, therefore, our experience emphasises the need, when planning a trial of cancer treatment, to give serious consideration first of all to the number of events required in order to obtain sufficient power to detect the percentage difference that will satisfy clinicians who will have to base any change in their method of treatment on the trial results. It is particularly important not to underestimate this number in a trial where the aim is to test therapeutic equivalence. Having decided on this number, then existing data on the pattern of deaths and recurrences in the disease should be used to determine the maximum follow-up time on each patient and the time for definitive analysis. Little will be gained by follow-up beyond the time when the event-free curves are beginning to flatten out, since the few extra events that will be recorded beyond this point contribute little to the precision of the estimates of treatment differences. Our trial has provided data on the pattern of deaths, local recurrences and late effects on normal tissue following the treatment of squamous cell carcinoma of the laryngopharynx, and these data can now be used as a guide in the conduct of future trials at this site. Had such data been available to us in 1962, when planning the trial, then it is clear that, instead of delaying our final definitive analysis until more than 20 years after the first patient entered the trial, we could have achieved an almost equivalent result 7–8 years earlier.

### Appendix

*Relative risk, log relative risk and difference in event-free rates*

The relative risk, $R$, is the ratio of the hazard rate in one group to that in the other group and is related to the log relative risk, $\Delta$, by the equation:

$$R = e^{\Delta}$$

The standard error of $\Delta$ is $V^{-\frac{1}{2}}$ where $V^{-1} \simeq E_1^{-1} + E_2^{-1}$ (Haybittle & Freedman, 1979), $E_1$ and $E_2$ being the expected events on the null hypothesis in each arm of the trial. With approximately equal numbers of patients in each arm and $\Delta$ not very different from zero:

$$E_1 = E_2 = E, \text{ and } V^{-1} = 2E^{-1} = 4d^{-1}$$

since $d$, the total number of events, equals $E_1 + E_2 = 2E$. Thus $V^{-\frac{1}{2}} = 2d^{-\frac{1}{2}}$ and the 95% confidence interval on an estimate of $\Delta$ is from $\Delta - 2 \times 1.96d^{-\frac{1}{2}}$ to $\Delta + 2 \times 1.96d^{-\frac{1}{2}}$, i.e. the confidence limits are $\pm 3.92d^{-\frac{1}{2}}$.

If $\Delta$ is small, then $R \simeq 1 + \Delta$, so that a log relative risk of 0.20 corresponds to a relative risk of 1.20 (more accurately 1.22 if no approximation is made). Thus confidence limits $\pm x$ on the estimate of log relative risk as shown in Figure 3 correspond to very similar confidence limits on the relative risk, when $x$ is small. However, while the confidence interval on the former is set symmetrically about the estimate, the interval is not set symmetrically about the latter, and it is for this reason that the confidence limits for $\Delta$ rather than for $R$ are plotted in Figure 3.

A relative risk can be translated into a difference in event-free rates at a given time by using the equation:

$$P_2 = (P_1)^R$$

where $P_1$ and $P_2$ are the event-free rates at that time in groups 1 and 2 respectively and $R$ is the relative risk, group 2 to group 1. The relationship does not depend on the form of the event-free curve but only on the proportional hazards assumption, i.e. that $R$ remains constant over the time studied. For any given values of $P_1$ and $R$, $P_2$ can be calculated, and hence the difference, $P_1 - P_2$. Table I shows the relative risks that would give rise to three values of these differences, expressed as percentages, for three values of $P_1$.

**Table I** Relative risk (log relative risk) corresponding to differences (%) in event-free rates

| *Difference (%)* | $P_1$ | | |
|---|---|---|---|
| $P_1 - P_2$ | *25%* | *50%* | *75%* |
| 5 | 1.16 (0.15) | 1.15 (0.14) | 1.24 (0.21) |
| 10 | 1.37 (0.31) | 1.32 (0.28) | 1.50 (0.40) |
| 15 | 1.66 (0.51) | 1.51 (0.42) | 1.78 (0.57) |

For the example in the text of a 10% difference centred on 55%, i.e. a difference between 60% and 50%, the relative risk is 1.357.

We are grateful to the British Institute of Radiology and the Medical Research Council for financial support.

### References

ARMITAGE, P., McPHERSON, C.K. & ROWE, B.C. (1969). Repeated significance tests on accumulating data. *J. R. Stat. Soc. A*, **132**, 235.

BLACKWELDER, W.C. (1982). 'Proving the null hypothesis' in clinical trials. *Contr. Clin. Trials*, **3**, 345.

FREEDMAN, L.S. (1982). Tables of the number of patients required in clinical trials using the logrank test. *Stat. Med.*, **1**, 121.

GEORGE, S.L. & DESU, M.M. (1974). Planning the size and duration of a clinical trial studying the time to some critical event. *J. Chron. Dis.*, **27**, 15.

HAYBITTLE, J.L. (1971). Repeated assessment of results in clinical trials of cancer treatment. *Br. J. Radiol.*, **44**, 793.

HAYBITTLE, J.L. & FREEDMAN, L.S. (1979). Some comments on the logrank test statistic in clinical trial applications. *Statistician*, **28**, 199.

MACHIN, D. & CAMPBELL, M.J. (1987). *Statistical Tables for the Design of Clinical Trials*. Blackwell Scientific Publications: Oxford.

MAKUCH, R. & SIMON, R. (1978). Sample size requirements for evaluating a conservative therapy. *Cancer Treat. Rep.*, **62**, 1037.

PETO, R., PIKE, M.C., ARMITAGE, P. & 7 others (1977). Design and analysis of randomized clinical trials requiring prolonged observation of each patient. II. Analysis and examples. *Br. J. Cancer*, **35**, 1.

POCOCK, S.J. (1977). Group sequential methods in the design and analysis of clinical trials. *Biometrika*, **64**, 191.

RODARY, C., COM-NOUGE, C. & TOURNADE, M. (1989). How to establish equivalence between treatments: a one-sided clinical trial in paediatric oncology. *Stat. Med.*, **8**, 593.

WIERNIK, G., BATES, T.D., BERRY, R.J. & 15 others (1982). Seventh interim progress report of the British Institute of Radiology fractionation study of 3F/week versus 5/week in radiotherapy of the laryngo-pharynx. *Br. J. Radiol.*, **55**, 505.

WIERNIK, G., BATES, T.D., BLEEHEN, N.M. & 10 others (1990). Report of the general clinical results of the British Institute of Radiology fractionation study of 3F/week versus 5F/week in radiotherapy of carcinoma of the laryngo-pharynx. *Br. J. Radiol.*, **63**, 169.